# Current Evidence on Safety, Efficacy and Efficiency of Sublingual Vaccine Uromune^®^ in Prevention of Recurrent Urinary Tract Infections: A Literature Review

**DOI:** 10.3390/pathogens15010042

**Published:** 2025-12-30

**Authors:** José Emilio Hernández-Sánchez, María Fernanda Lorenzo-Gómez, Carmen González-Enguita

**Affiliations:** 1 General University Hospital of Villalba, 28400 Madrid, Spain; 2 University Hospital of Salamanca, 37007 Salamanca, Spain; 11948872g@saludcastillayleon.es; 3 Fundación Jiménez Díaz University Hospital, 28040 Madrid, Spain; cgenguita@fjd.es

**Keywords:** UTI, sublingual vaccine, immunoprophylaxis, antimicrobial resistance, public health, cost-effectiveness

## Abstract

Recurrent urinary tract infections (rUTIs) are a prevalent public health problem in women, with significant clinical, psychological, and economic consequences. Standard antibiotic prophylaxis, while effective, is limited in the medium term due to the risk of bacterial resistance and potential side effects. This narrative review summarizes the current evidence on the efficacy, safety, and economic impact of Uromune^®^—a sublingual bacterial vaccine—as a preventive strategy for rUTIs. A literature search was conducted, focusing on systematic reviews and meta-analyses that evaluated Uromune^®^ in women and in other specific at-risk populations. Available data show that Uromune^®^ reduces the frequency of UTI episodes, prolongs recurrence-free intervals, and decreases overall antibiotic use. The vaccine has a favorable safety profile, with predominantly mild adverse effects and a low discontinuation rate. Furthermore, its use has been associated with improved quality of life and a marked reduction in direct and indirect healthcare costs. These findings support Uromune^®^ as an effective, safe, and potentially cost-effective alternative to conventional antibiotic prophylaxis in the current context of increasing antimicrobial resistance.

## 1. Introduction

Recurrent urinary tract infections (rUTIs) affect many women and cause significant physical and psychological discomfort [1]. Although antibiotics remain the standard treatment, they are associated with adverse effects, high costs, and the risk of bacterial resistance. Therefore, non-antibiotic alternatives have been explored, among which immunoprophylaxis with the sublingual vaccine Uromune^®^ stands out. Studies have shown that this vaccine is safe, reduces the incidence of UTIs, decreases antibiotic use, improves quality of life, and has a positive economic impact [2].

Uromune^®^ differs from other immunoprophylactic strategies through its multivalent composition and broad bacterial spectrum, which is difficult to match by other preparations, many of which only include *E. coli* or use less controlled manufacturing methods. It is also unique due to its underlying immunological mechanism, clinical evidence of efficacy and safety with persistent beneficial effects, and its relevance regarding the global crisis of antibiotic resistance.

The data suggest that this immunotherapy approach is effective. In fact, most patients remain recurrence-free for the first year, with benefits that can be maintained for several years after immunization.

## 2. Materials and Methods

A narrative review of the scientific literature was conducted to synthesize and analyze the available evidence on the clinical utility of a sublingual vaccine (Uromune^®^) in preventing rUTIs, especially in women.

The literature search was conducted in the PubMed/MEDLINE databases. Combinations of MeSH terms and keywords were used, such as “recurrent UTI,” “sublingual vaccine,” “immunoprophylaxis,” “antimicrobial resistance,” “public health,” and “cost-effectiveness.”

Primary studies, systematic reviews, meta-analyses, clinical trials, and observational studies published in English and Spanish from 2000 to the present were included. The results of the selected studies were analyzed descriptively. The main findings regarding short- and long-term clinical efficacy, reduction in UTI episodes, safety profile, and application in clinical subgroups were highlighted, and reported cost-effectiveness analyses were reviewed.

No meta-analysis was performed, but a qualitative synthesis of the most relevant results from the existing literature was conducted, with special emphasis on systematic reviews and comparative studies that included data on the sublingual vaccine (Uromune^®^).

We believe a narrative review is preferable when seeking to expand knowledge and contextualize the preventive role of immunoprophylaxis with a sublingual vaccine because it allows us to integrate and explain available evidence from different clinical and scientific perspectives, especially in emerging areas with heterogeneous studies. In contrast, a systematic review focuses solely on quantifying effects from an exhaustive and protocolized search, making it less flexible to address nuances, controversies, and contextualized clinical application.

## 3. Relevant Sections

### 3.1. rUTIs as a Public Health Issue

Uncomplicated urinary tract infections (UTIs) are common among women. In fact, all women will experience at least one episode during their lifetime, most of which are uncomplicated and easily resolved with antibiotics [1,3]. The recurrence rate is 2.6 (0.3–7.6) episodes per patient/year, with a 24% risk of recurrence at 6 months, rising to 70% within one year [4,5]. The annual incidence of rUTIs—defined as ≥3 episodes per year or ≥2 in 6 months—is approximately 3% [4]. Women with rUTIs experience a significant reduction in quality of life, often presenting with psychological and sexual dysfunctions, along with loss of workdays and disruption of daily activities [6,7,8,9].

Recurrent ascending infections or persistent infections are underlying pathophysiological mechanisms in the origin of rUTIs. *Escherichia coli* strains are responsible for 52–77% of cases, particularly serotypes O4, O6, and O75. *E. coli* can replicate intracellularly and form intracellular bacterial communities (IBCs), which may remain dormant during antibiotic therapy. Disruption of these IBCs can lead to recurrence. Uropathogenic *E. coli* can enter a state of intracellular quiescence because it becomes sequestered in endosomal vesicles of urothelial cells. In this state, metabolism is greatly reduced and it does not replicate, trigger an immune response, or become effectively targeted by antibiotics that act on dividing bacteria. These quiescent bacteria constitute intracellular reservoirs that can later reactivate, rupture the cell, or be released exocytically, leading to new episodes of UTI with the same strain [10,11].

Both therapeutic and preventive management of rUTIs in women often involves antibiotics—either for treating acute episodes, for long-term or postcoital prophylaxis, or as part of self-treatment protocols. Approximately 25% of all antibiotic prescriptions are for UTIs [12]. However, antibiotic use can lead to adverse events that reduce adherence to treatment, some of which, although infrequent, are serious or potentially life-threatening, such as toxic epidermal necrolysis and Stevens–Johnson syndrome. Special mention should be made of the potential selection of multidrug-resistant pathogens capable of causing severe infections that are difficult to treat, with a high risk of mortality and economic burden. UTIs caused by pathogens other than *Escherichia coli* often occur in complex clinical settings that favor colonization by multidrug-resistant flora and reduce the effectiveness of standard treatment regimens. Microorganisms such as *Klebsiella*, *Pseudomonas*, *Proteus*, and *Enterococcus* frequently exhibit advanced resistance mechanisms and high ability to form biofilms, which limits the action of oral antibiotics administered in short courses. These characteristics necessitate the use of longer, intravenous, or broader-spectrum treatments, sometimes in combination, along with the correction of predisposing factors, to achieve clinical resolution and reduce the risk of recurrence. Microbiological findings following sublingual administration of Uromune^®^ show a simplification of urinary bacterial flora, with a significant reduction in polymicrobial urine cultures and a decrease in pathogens typical of complex cases [13,14,15,16,17].

There are limited data regarding uropathogen resistance in randomized controlled trials (RCTs) focused on UTI prevention and antibiotic prophylaxis. In a meta-analysis of six RCTs involving patients under 18 years of age with vesicoureteral reflux (VUR) and rUTIs, Selekman et al. reported that patients receiving prophylaxis were 6.4 times (2.7–15.6) more likely to develop multidrug-resistant infections [18].

### 3.2. Economic Impact of UTIs

An estimated 150 million people worldwide—primarily women—suffer from UTIs each year. In the United States alone, it is estimated that 11.3 million women experience UTIs annually, accounting for 7 million outpatient visits, 2 million emergency room visits, and 350,000 hospitalizations. This leads to significant healthcare expenditures, amounting to approximately USD 3.5 billion annually, with health-related costs being 1.4 times higher in women with UTIs compared to those without [19,20,21,22,23,24].

rUTI management is associated with substantial direct and indirect costs. Direct costs include medical consultations, diagnostic testing, pharmacological prescriptions, and hospitalizations. These vary considerably between countries, ranging from EUR 229 per year in Italy to EUR 1001.1 per year in Spain, with the largest proportion attributed to primary care visits [2,25,26,27].

Regarding indirect costs, the number of hours lost from daily activities and time spent bedridden following a cystitis episode can exceed 20 h, reaching over three days per year in cases of rUTIs. In France, it is estimated that 9% of women with UTIs require medical leave, with an average duration of 2.39 days, resulting in total indirect costs of EUR 13.9 million. On average, each UTI in premenopausal women is associated with 6.1 days of disability and 2.5 days of absence from work or school [3]. If the infection is caused by a resistant strain, it is associated with longer symptom duration, more time spent bedridden, more missed workdays, and increased disruption of daily life [8,28,29].

A continuous increase has been observed in the isolation of antibiotic-resistant pathogens. In Spain, an eightfold increase has been reported in the proportion of extended-spectrum beta-lactamase (ESBL)-producing *E. coli* isolates. In the European Union, 25,000 deaths are attributed annually to resistant bacterial infections, with an additional 2.5 million hospital days, amounting to an estimated EUR 1.5 billion in annual healthcare costs. A similar scenario is seen in the United States, where additional costs are estimated at USD 20–25 billion in healthcare expenditures and USD 35 billion in lost productivity—totaling USD 60 billion in additional expenses [30,31].

rUTIs represent a common issue among women, generating significant economic burden and impacting quality of life. Gaitonde and Wilke estimated the initial costs of rUTIs to be EUR 730 and EUR 336.34 (95% CI: €114.07–978.42), primarily due to the need for additional diagnostic tests, more frequent visits to urologists, and increased antibiotic requirements. These costs are even higher when multidrug-resistant pathogens are involved, as they often require prolonged intravenous antibiotic treatment (10–14 days), with additional costs of USD 3390 [26,32].

### 3.3. Preventive Alternatives to Antibiotics in rUTIs

Patients with rUTIs often present complex challenges. It is common to encounter individuals who express distrust or dissatisfaction with how healthcare professionals handle their condition, leading some to pursue inadvisable alternatives [9,33].

Both American and European clinical practice guidelines include non-antibiotic recommendations for managing rUTIs, such as the use of vaginal estrogens in postmenopausal women, probiotics, cranberry extracts, D-mannose, and increased fluid intake. However, evidence for these strategies is not robust due to limitations in methodological quality, sample size, heterogeneity of interventions, and inconsistent results across studies and meta-analyses. Phages and postbiotics are currently considered experimental therapies outside the scope of standard recommendations [16,34].

In contrast, immunoprophylaxis with the sublingual vaccine Uromune^®^ has emerged as a safe and effective preventive alternative that reduces the risk of rUTIs. Comparative studies and meta-analyses have shown positive outcomes, even when compared directly with antibiotics [6,15,35].

### 3.4. Uromune^®^ Composition and How It Works

Uromune^®^ (Inmunotek S.L., Alcala de Henares, Spain) is a mucosal bacterial vaccine against rUTIs administered via sublingual spray. It is currently approved and available in 26 countries. The treatment consists of two daily 100 µL sublingual sprays for a duration of 3 months [36,37,38,39].

The MV140 formulation is a suspension containing four common uropathogens in equal proportions: *Escherichia coli* (25%), *Klebsiella pneumoniae* (25%), *Enterococcus faecalis* (25%), and *Proteus vulgaris* (25%). In some countries, such as Spain, the vaccine can be customized with patient-specific bacterial strains obtained from a urine sample collected during an active infection [36,37,38,39].

The sublingual route used for Uromune^®^ is gaining interest compared to other mucosal immunization strategies as it avoids degradation by gastrointestinal fluids and induces both systemic and genitourinary tract-specific immune responses [36,37,38,39].

Immune dissemination to the effector site—the bladder mucosa—is achieved through the migration of lymphocytes and other immune cells from the inductive site (sublingual mucosa) via the mucosa-associated lymphoid tissue (MALT) [40].

During a UTI, the pathogen induces an inflammatory response in the urinary tract, usually resolved with antibiotics. However, in women with rUTIs, epithelial damage leads to an increased T-helper 2 (Th2) cell response, which diminishes the ability to respond to both exogenous pathogens and the endogenous urinary microbiota, thereby creating a favorable environment for recurrence [41].

Uromune^®^ stimulates antibody production and activates human dendritic cells to generate Th1, Th17, and IL-10-producing helper T cells, resulting in anti-inflammatory T cell responses both in secondary lymphoid organs and locally in the bladder [36,42]. The induction of adaptive immunity likely underlies the clinical protection observed after treatment cessation. Additionally, trained immunity—defined as enhanced responsiveness of innate immune cells—may contribute to a heightened ability to respond to subsequent bacterial challenges [43].

### 3.5. Analysis of Evidence Supporting Use of Uromune^®^

Several studies have evaluated the efficacy of Uromune^®^ in the prevention of rUTIs. However, aside from published meta-analyses, many studies have methodological limitations as they are predominantly retrospective, open-label, and non-randomized studies lacking control groups, with unclear recruitment strategies and imprecise definitions of UTI-free status—factors that may introduce potential bias. An additional problem is the possible overlap of the studies included in the reviews.

In addition to the sublingual vaccine Uromune^®^, other vaccine forms include OM-89/UroVaxom (oral tablet), Solco-Urovac (vaginal suppository/intramuscular injection), and ExPEC4V (intramuscular injection). While all appear to be effective, Uromune^®^ has shown superior outcomes in several studies [44]. Table 1 (cited in Prattley) summarizes the main characteristics of these vaccines.

Prattley’s systematic review and meta-analysis of 17 studies (3228 patients: 1970 in the vaccine group and 1258 in the comparison group), including Uromune^®^ (3 studies), OM-89/UroVaxom [9], Solco-Urovac [4], and ExPEC4V [1], reports a significant short-term odds ratio (OR) of 0.17 (95% CI: 0.06–0.50) for immunoprophylaxis, with Uromune^®^ demonstrating the most significant effect at 6 months. The UTI-free rate with vaccination ranged from 63.5% to 81% compared to 3–5.6% in the antibiotic prophylaxis group [44].

In the same study, long-term efficacy (>6 months) showed a significant overall OR of 0.20 (95% CI: 0.07–0.59). However, when Uromune^®^ was excluded from the analysis, the effect lost significance (OR: 0.66; 95% CI: 0.35–1.26). The median time to UTI recurrence was 180 days with Uromune^®^ compared to 19 days with antibiotic prophylaxis. The long-term UTI-free rate with Uromune^®^ ranged from 56.6% to 90.3%, with the longest-lasting effect reported at 15 months (56.6%). In contrast, nearly all patients receiving antibiotic prophylaxis experienced at least one UTI at 12 or 15 months. The heterogeneity and quality of the included studies limit the conclusions and increase the risk of bias, potentially overestimating the real benefit of vaccines in rUTIs, without resolving key issues such as the duration of effect or efficacy in at-risk populations compared to standard antibiotic prophylaxis [44].

Mak’s systematic review and meta-analysis of 14 studies (2822 patients) investigated the protective role of five vaccines (Strovac, Uro-Vaxom, ExPEC4V, Uromune^®^, and Solco-Urovac). Compared to the placebo, the pooled relative risk (RR) was 1.52 (95% CI: 1.05–2.20), indicating that vaccinated patients had an approximately 50% higher chance of remaining UTI-free in the short term. The number needed to treat (NNT) was 6.45 (95% CI: 2.80–64.80). Importantly, all four studies evaluating Uromune^®^ reported significant benefits [35].

A subgroup analysis by vaccine type indicated that short-term efficacy varied by product, with Uromune^®^ showing a clear advantage. However, subgroup analysis was performed only for Solco-Urovac and Uro-Vaxom. Lorenzo-Gómez et al. reported an RR of 2.23 (95% CI: 1.43–3.47), the largest effect size among all included studies. The meta-analysis has limitations due to heterogeneity between studies and possible publication bias, so the results should be interpreted with caution and considered indicative [35].

In the same meta-analysis by Mak [35], studies by Lorenzo-Gómez [45,46] reported significantly higher UTI-free rates (56.6–90.3%) in women receiving sublingual Uromune^®^ at 9 and 12 months compared to those on antibiotic prophylaxis. Notably, 34.6% of women in the Uromune^®^ group remained UTI-free at 15 months, while none in the antibiotic group did so at 12 or 15 months. Both studies are observational without randomization and blinding, which impacts the internal validity and strength of the conclusions, so they should be considered preliminary and hypothesis-generating evidence. Table 2 presents the main studies from Mak’s review [45,46,47,48].

Nickel’s systematic review [49] of five studies involving 1408 women with rUTIs treated with sublingual Uromune^®^ suggests that it may be a safe alternative to antibiotic prophylaxis. In particular, 519 women who received daily vaccination for 3 months achieved significantly higher UTI-free rates (35–90%) than those treated with antibiotic prophylaxis for 6 months (*p* < 0.001). UTI-free rates in the Uromune^®^ group ranged from 33% to 78% over a follow-up period of 9 to 24 months [15]. The heterogeneity of the included studies, almost exclusively non-randomized observational studies, implies a high risk of bias, which means that the efficacy rates should be interpreted with caution as preliminary data. Table 2 presents the key studies from Nickel’s review [45,46,50,51].

These findings are consistent with another study by Nickel conducted in the Health Canada context, which reported an 82% reduction in UTI rates during the 9-month period following vaccination, decreasing from 11.5 to 2.1 UTIs/month [6].

In a multicenter, randomized, double-blind, placebo-controlled European clinical trial involving 240 women with rUTIs, Lorenzo-Gómez reported a significant reduction in the median number of UTIs from 3 to 0 during a 9-month efficacy period in those receiving MV140 for 3 or 6 months. The median time to the first UTI was 275 days [IQR: 87.0–275.0] compared to 48.0 days in the placebo group. This trial justifies considering MV140 as a safe and very promising non-antibiotic preventive option in women with uncomplicated UTIs. However, the lack of direct comparisons with standard antibiotic prophylaxis and the short follow-up period suggest that caution is needed regarding the strength of the recommendation, and confirmation is required with trials that include more patients and a longer time horizon [53].

A subanalysis found that vaccinated groups had significantly fewer general UTI symptoms, fewer days of antibiotic use, and improved quality of life (SF-36), demonstrating a reduction in the personal burden of disease in women with rUTIs. This observed reduced burden of disease is consistent with a lower recurrence of UTIs but should be interpreted as an exploratory and confirmatory signal, pending replication in other clinical trials [54].

Other studies by Yang, Ramírez-Sevilla, and Carrión-López also report variable UTI-free rates after vaccination, with protection decreasing over time—especially in patients with risk factors. These studies are observational and unblinded with a high risk of bias. Although they support the plausibility and clinical interest of immunoprophylaxis, they should be considered preliminary evidence [50,51,52].

### 3.6. Utility of Uromune^®^ in Other Patient Populations

The efficacy of sublingual Uromune^®^ in preventing rUTIs is not limited to women with uncomplicated infections. Its usefulness has been reported in various other clinical settings.

Several studies suggest that Uromune^®^ may be effective in reducing UTIs in men, with UTI-free rates ranging from 38 to 50% at 3 months, from 30 to 44.8% at 6 months, and 37.9% at 9 months. Some reports even indicate UTI-free rates as high as 71% at one year [50,51].

Uromune^®^ has also demonstrated efficacy in patients with chronic bacterial prostatitis, with significant improvements reported in SF-36 questionnaire scores. Furthermore, by avoiding prolonged antibiotic therapy, Uromune^®^ may help prevent the development of resistant bacterial strains in this population, which is often treated empirically due to difficulty in identifying the causative pathogen [55].

Patients with neurogenic bladders, who have a high incidence of rUTIs, have shown reduced annual UTI rates, increased UTI-free intervals, fewer hospitalizations, and improved quality of life with sublingual Uromune^®^ administration [56].

In patients with rheumatic diseases treated with immunomodulatory biologics and suffering from rUTIs, Uromune^®^ has shown to significantly reduce the number of UTI episodes (*p* < 0.001) compared to the previous year, along with reduced antibiotic prescriptions and fewer unscheduled medical visits [57,58].

rUTIs are also common in patients with chronic kidney disease. In this population, a 3-month course of Uromune^®^ was effective, with 26.9% of patients remaining UTI-free and 73.1% experiencing fewer episodes. Efficacy was greater in those with better renal function [59].

Among kidney transplant recipients, Uromune^®^ administration led to a reduction in annual UTI episodes from 4.2 to 2.7 (*p* < 0.001), with 46.5% experiencing fewer episodes and 16.3% remaining completely free of infection. This was achieved via a humoral immune response without adverse effects on renal function or the development of new anti-HLA antibodies [60].

In institutionalized elderly patients—both men and women—Uromune^®^ has been associated with a reduction in UTI episodes and improved quality of life compared to antibiotic prophylaxis. In women, the median UTI rate dropped to 0.1 UTIs/month, with 18% remaining UTI-free for 12 months and 81.7% receiving treatment for fewer than three UTIs per year. Sublingual administration of the vaccine is simple and well-suited for this patient population [61].

In women with stress urinary incontinence treated with suburethral sling procedures who also suffer from rUTIs, polyvalent bacterial vaccination has shown to be more effective than suppressive or on-demand antibiotic regimens. Uromune^®^ may also be effective in this group, given that its immunomodulatory mechanism is independent of the underlying anatomical cause of rUTIs [62,63].

### 3.7. Review of the Role of Revaccination

Mak’s systematic review and meta-analysis of 14 studies (2822 patients), which assessed the protective role of five vaccines (Strovac, Uro-Vaxom, ExPEC4V, Uromune^®^, and Solco-Urovac), identified three studies that support the use of booster regimens, specifically with Solco-Urovac compared to placebo, reporting a pooled RR of 2.74 (95% CI: 2.16–3.48) in favor of vaccination [35].

One study explored the potential benefits of revaccinating with Uromune^®^ 18 months later, concluding that it is not only safe but may also offer additional protection for patients with rUTIs [60].

Ramírez-Sevilla also suggested that administering second and third cycles of Uromune^®^ maintains its effectiveness while preserving tolerability, reporting UTI-free rates of 79.1% and 69.3% and rates of 76.1% and 66.5% at 3 and 6 months, respectively. The article does not specify precisely when the second or third cycle was initiated in each patient. There is no standardized protocol or firm consensus regarding the second or third cycle [51].

### 3.8. Safety Profile of Uromune^®^

Available evidence indicates that Uromune^®^ has a favorable safety profile in women with rUTIs. The incidence of adverse events is low (0–13%), all classified as Clavien-Dindo grades I–II, and treatment discontinuation is rare. In fact, Mak’s systematic review and meta-analysis found no difference in adverse event incidence between Uromune^®^ and placebo (RR: 0.96; 95% CI: 0.64–1.46). The most common side effects reported include headache, gastrointestinal disturbances, dizziness, flu-like symptoms, and skin rash. No cases of death or hospitalization have been reported [6,35,44,50,51,52,53].

Patient satisfaction with Uromune^®^ is high. Its simple and painless administration contributes to good adherence, with lower dropout rates compared to other vaccines, such as the 3.5% dropout observed with UroVaxom [50].

There are no clinical data or controlled studies evaluating the efficacy or safety of this medication in pregnant women, so its use is generally not recommended in this population.

### 3.9. Cost-Effective Analysis of Uromune^®^ in a Scientific Context

Uromune^®^ has demonstrated a significant reduction in UTI episodes and antibiotic use, which may translate into lower healthcare resource utilization and reduced risk of bacterial resistance, both in individual patients and at the population level.

In a study with a mean follow-up of 1.71 years involving 166 women with rUTIs, Carrión López P reported a significant reduction (*p* < 0.0001) in the number of UTI episodes (from 6.19 to 2.81), urine cultures (from 4.27 to 2.67), ultrasounds (from 0.37 to 0.12), antibiotic packages consumed (from 7.34 to 2.89), primary care visits (from 4.69 to 2.05), emergency visits (from 0.41 to 0.13), and hospital admissions (from 10.2% to 2.8%). Overall, a significant reduction (*p* < 0.0001) in total average annual cost per patient was observed, decreasing from EUR 1001.1 to EUR 669.1 (EUR 497.1 + EUR 172.38 if the cost of the vaccine is included) [2,52].

In a prospective study involving 377 patients with rUTIs who received antibiotic prophylaxis (126), autovaccine (126), or the polyvalent bacterial vaccine MV140 with selected strains (126), Ramírez-Sevilla reported that both the autovaccine (80.8%; 61.6%; EUR 20,763.73) and MV140 (81.7%; 74.6%; EUR 18,866.14) were more effective in reducing the number of UTI episodes to 0–1 at 3 and 6 months, with lower healthcare costs during follow-up compared to antibiotic prophylaxis (65%; 44%; EUR 21,171.87). Additionally, MV140 prepared with selected strains appeared to be more effective and less costly than the autovaccine [48].

In our recently published study involving 1614 women receiving prophylaxis for rUTIs (444 on antibiotic prophylaxis, 732 on Uromune^®^, and 438 on other preventive measures), we found that Uromune^®^ was more cost-effective than the alternatives, with greater reductions in primary care visits, follow-up urology appointments, UTI episodes, and the need for urine analyses, cultures, CT scans, and ultrasounds. These findings underscore the transformative potential of immunoprophylaxis as a sustainable and cost-effective alternative to traditional antibiotic prophylaxis, especially in light of the current challenges posed by antimicrobial resistance [64]. Table 3 presents the main studies conducted on Uromune^®^ from an economic perspective [2,48,52,64].

Microbiological findings following sublingual administration of Uromune^®^ show a simplification of the urinary bacterial flora, with a significant reduction in polymicrobial urine cultures and a decrease in pathogens typical of complex cases, such as *Enterococcus faecalis* and *Proteus mirabilis*, which practically disappear. Recurrences that persist are concentrated in *Escherichia coli* and, secondarily, *Klebsiella* spp., which then constitute most isolates, resulting in a less frequent, less polymicrobial, and microbiologically more homogeneous pattern of recurrent UTIs after vaccination [14]. This simplification of the flora, with fewer polymicrobial and multidrug-resistant episodes, suggests a likely decrease in hospital admissions for complicated UTIs, which will reduce both the cost per episode and the cost associated with antimicrobial resistance, a significant component of the economic burden of rUTIs.

### 3.10. Limitations of the Review Article

The main evidence indicates that immunoprophylaxis with sublingual vaccine can reduce the recurrence of urinary tract infections and antibiotic use, showing promising results in recent clinical studies. However, these findings should be interpreted with caution due to the methodological limitations of the narrative review, including the lack of systematic criteria for study selection and analysis, potential bias, and limited external validity. This makes it necessary to consider the need for controlled and comparative trials to confirm its effectiveness and clinical applicability.

## 4. Conclusions and Future Directions

Uromune^®^ is a safe, effective, and efficient alternative for the prevention of recurrent urinary tract infections in women. While additional evidence is needed, it may also be useful in other clinical scenarios.

There are several areas that require further scientific investigation. A key priority is conducting clinical trials without methodological limitations, which would allow for even more robust validation of the efficacy and safety observed in recent observational studies and meta-analyses. Comprehensive pharmacoeconomic analyses should be incorporated, especially in resource-limited healthcare settings.

The long-term immunological effect of Uromune^®^ and the role of revaccination in different patient profiles should also be explored.

Mechanistic research is needed to delve deeper into the underlying immunological processes induced by the vaccine and identify biomarkers of clinical response, thereby optimizing their personalized use.

## Figures and Tables

**Table 1:** Main characteristics of vaccines available for rUTI, as mentioned in the systematic review and meta-analysis by Prattley [44].

Vaccine Type	Administration Route	Composition
ExPEC4V	Single intramuscular injection	Attenuated exotoxin A from *Pseudomonas aeruginosa* + surface antigens of 4 *E. coli* serotypes
Uromune^®^	2 sublingual sprays/day for 3 months	*MV140:* Inactivated suspension of *E. coli*, *K. pneumoniae*, *E. faecalis*, *P. vulgaris* *Spain:* Patient-specific preparation based on urine sample
UroVaxom (OM-89)	1 oral tablet/day for 3 months. Booster possible in months 6–9	Lysate of 18 *E. coli* strains
Solco-Urovac	Weekly vaginal suppository, then monthly + weekly IM injection up to month 6	10 strains of *E. coli*, *K. pneumoniae*, *Proteus mirabilis*, *Proteus morganii*, and *E. faecalis*

**Table 2:** Main studies from Mak’s [35] and Nickel’s [49] review.

Author	Study Type	Primary and Secondary Outcomes	Type of Prophylaxis	Male vs. Female	Mean Age (Years)	% UTI-Free	Follow-Up Period	Risk of Bias
Lorenzo-Gómez (2013) [46]	Retrospective cohort study	UTI-free patients and time until new UTI Number of positive urine cultures and antibiotic resistance	Uromune^®^ MV140 3 months (159) vs. AB 6 months (160)	100% female	47.7 vs. 48.1	63.5% vs. 5.6% (3 months) 56.6% vs. 2.5% (9 months) 34.6% vs. 0% (15 months)	15 months	Retrospective design, not randomized and no blinding
Lorenzo-Gómez (2015) [45]	Retrospective cohort study	Time to UTI and proportion of UTI-free patients Risk reduction, NNT, and bacterial resistance	Uromune^®^ MV140 3 months (360) vs. AB 6 months (339)	100% female	60 vs. 59	90.3% vs. 0% (12 months)	12 months	Retrospective design, not randomized and no blinding
Wagenlehner (2015) [47]	Randomized controlled trial crossover	UTI number Tolerability and safety	Uro-Vaxom (220) vs. placebo + AB (231)	100% female	4 4.4 vs. 43.4	47.7% vs. 48.5% (*p* > 0.05)	6 months	High protocol violations and low incidence of UTI during the study
Yang (2018) [50]	Open prospective cohort study	Time to first UTI recurrence Safety and side effects	Uromune^®^ MV140 3 months (75)	100% female	56	78.7% (12 months)	12 months	Absence of a control group and lack of randomization
Ramírez-Sevilla (2019) [51]	Prospective descriptive non-randomized study	Number UTIs at 3 and 6 months Safety and outcomes according to patient profile	Uromune^®^ MV140 3 months (648)	17.3% vs. 82.7%	73.5 vs. 59	45.4% (3 months) 32.7% (6 months)	6 months	Absence of a control group and lack of randomization
Carrión-López (2020) [52]	Quasi-experimental pretest–posttest study	Reduction in number of UTIs Reduction in positive urine cultures, antibiotic needs, and symptoms	Uromune^®^ MV140 3 months (166)	100% female	62.3	74.4% (3 months) 68.1% (6 months) 52.4% (12 months) 44.5% (24 months)	24 months	Absence of a control group
Ramírez-Sevilla (2023) [48]	Prospective cohort study	Number of UTIs at 3 and 6 months Healthcare costs and demographic characteristics	Uromune^®^ MV140 and autovacuna (252) Vs. AB (126)	100 % female	71	23% vs. 11.9%	12 months	Non-random assignment of patients to groups

**Table 3:** The main studies conducted on Uromune^®^ from an economic perspective [2,48,52,64].

Author	Study Type	Type of Prophylaxis	Follow-Up Period	Patient Characteristics	Clinical Results	Economic Results	Main Conclusion
Carrión López (2020 y 2022) [2,52]	Quasi-experimental pretest-posttest study	Uromune^®^ MV140	1.71 years	166 women	Significant reduction in UTI episodes, urine cultures, ultrasounds, primary care and emergency visits, and hospitalizations	Total annual cost/patient: EUR 1001.1 to EUR 669.1 (EUR 497.1 + EUR 172.38 incl. vaccine); *p* < 0.0001	Uromune^®^ reduces UTI recurrence and health utilization
Ramirez Sevilla (2023) [48]	Prospective cohort study	MV140 vs. Autovaccine vs antibiotic prophylaxis	6 months	377 women	Higher percentage of patients free of UTIs compared to antibiotic prophylaxis	Mean cost: MV140 EUR 18,866.14 autovaccines EUR 20,763.73 antibiotics EUR 21,171.87	MV140 is more effective and cost-saving vs. autovaccine and antibiotic prophylaxis
Hernández-Sánchez (2025) [64]	Retrospective observational study with cost-effectiveness economic analysis	Uromune^®^ Autovaccine vs. antibiotic prophylaxis vs. other preventive measures	12 months	1614 women	Reduction in UTI episodes, primary care and urology consultations, and need for urine cultures, CT scans, and ultrasounds	Higher cost-effectiveness compared to other prophylactic measures	Uromune^®^ is a cost-effective and sustainable alternative to conventional antibiotic prophylaxis

## Data Availability

The original contributions presented in this study are included in the article. Further inquiries can be directed to the corresponding author.
